# Frequency-Mode Study of Piezoelectric Devices for Non-Invasive Optical Activation

**DOI:** 10.3390/nano15211650

**Published:** 2025-10-29

**Authors:** Armando Josué Piña-Díaz, Leonardo Castillo-Tobar, Donatila Milachay-Montero, Emigdio Chavez-Angel, Roberto Villarroel, José Antonio García-Merino

**Affiliations:** 1 Departamento de Ingeniería en Control y Automatización, Escuela Superior de Ingeniería Mecánica y Eléctrica Unidad Zacatenco, Instituto Politécnico Nacional, México City 07738, Mexico; 2 Departamento de Mecánica, Facultad de Ingeniería, Universidad Tecnológica Metropolitana, José Pedro Alessandri 1242, Nuñoa, Santiago 7800002, Chile; 3 Departamento de Física, Facultad de Ciencias Naturales, Matemática y del Medio Ambiente, Universidad Tecnológica Metropolitana, Las Palmeras 3360, Ñuñoa, Santiago 7800003, Chile; 4 ANID–Millennium Science Initiative Program, Millennium Nuclei of Advanced MXenes for Sustainable Applications (AMXSA), Santiago 8320211, Chile; 5 Catalan Institute of Nanoscience and Nanotechnology, CSIC and BIST, Campus UAB, Bellaterra, 08193 Barcelona, Spain

**Keywords:** piezoelectric, piezo-optical effect, interferometric instrumentation, impedance spectroscopy

## Abstract

Piezoelectric materials are fundamental elements in modern science and technology due to their unique ability to convert mechanical and electrical energy bidirectionally. They are widely employed in sensors, actuators, and energy-harvesting systems. In this work, we investigate the behavior of commercial lead zirconate titanate (PZT) sensors under frequency-mode excitation using a combined approach of impedance spectroscopy and optical interferometry. The impedance spectra reveal distinct resonance–antiresonance features that strongly depend on geometry, while interferometric measurements capture dynamic strain fields through fringe displacement analysis. The strongest deformation occurs near the first kilohertz resonance, directly correlated with the impedance phase, enabling the extraction of an effective piezoelectric constant (~40 pC/N). Moving beyond the linear regime, laser-induced excitation demonstrates optically driven activation of piezoelectric modes, with a frequency-dependent response and nonlinear scaling with optical power, characteristic of coupled pyroelectric–piezoelectric effects. These findings introduce a frequency-mode approach that combines impedance spectroscopy and optical interferometry to simultaneously probe electrical and mechanical responses in a single setup, enabling non-contact, frequency-selective sensing without surface modification or complex optical alignment. Although focused on macroscale ceramic PZTs, the non-contact measurement and activation strategies presented here offer scalable tools for informing the design and analysis of piezoelectric behavior in micro- and nanoscale systems. Such frequency-resolved, optical-access approaches are particularly valuable in the development of next-generation nanosensors, MEMS/NEMS devices, and optoelectronic interfaces where direct electrical probing is challenging or invasive.

## 1. Introduction

Piezoelectric sensors play a crucial role in a wide range of technological, industrial, and biomedical applications, due to their ability to convert mechanical energy into electrical energy and vice versa. This effect, known as piezoelectricity, allows for highly sensitive and reliable measurements of pressure, vibration, force and acceleration in both sensing and actuation systems [1]. Among the available ceramic materials, lead zirconate titanate (PZT) remains one of the most widely used due to its high piezoelectric constant, excellent thermal stability, and ease of fabrication [2,3,4].

From a manufacturing perspective, piezoelectric sensors are often produced using methods such as powder metallurgy, which enable the fabrication of porous or granular ceramic materials with tailored properties to enhance piezoelectric performance. This method involves the compaction and sintering of fine powders, providing precise control over the microstructure and the resulting electromechanical response of the material [1]. The introduction of controlled porosity can improve certain dielectric properties and decrease the weight of the sensor. However, it may also compromise its mechanical robustness. Therefore, the degree of porosity must be optimized according to the specific requirements of the application, balancing sensitivity with structural robustness [2,3].

A fundamental aspect in designing and characterizing piezoelectric devices is the evaluation of their behavior in the frequency domain. The eigenmodes of these materials, corresponding to their resonant response to periodic excitation, are critical in applications such as ultrasonic transducers, resonators, and precision filters [4,5]. The efficiency and strength of these modes can be optimized through piezoelectric poling, a process that aligns the internal dipole domains under an applied electric field, thus amplifying their piezoelectric response [6]. Another key parameter that limits the performance is the mechanical quality factor (*Q_m_*), which reflects the energy lost due to internal mechanical damping of each vibrational modes [7]. Accurate characterization of *Q_m_* is critical to the selection of piezoelectric materials for high-power ultrasonic devices, where energy efficiency and mechanical robustness are essential [8,9].

Beyond traditional electrical characterization, optical instrumentation has emerged as a promising, non-invasive alternative for probing electromechanical responses. Optical techniques, such as interferometry offer high spatial and temporal resolution, and the ability to analyze devices under extreme or contact-restricted conditions. However, these methods also present limitations, including sensitivity to environmental noise, complex alignment procedures, and higher implementation costs compared to conventional electrical approaches [10]. Recent studies have demonstrated the successful use of optical methods to characterize piezoelectric sensors, enabling high-resolution detection and visualization of dynamic deformation patterns and internal stress distributions that are difficult to capture with contact-based techniques [11].

Furthermore, the integration of piezoelectric materials with optical activation schemes offers an attractive route for the wireless operation and control of sensors, actuators, and microelectromechanical systems (MEMS). In particular, high-intensity laser pulses can induce mechanical and electrical responses in piezoelectric materials, either through direct photoinduced polarization or indirectly via photothermal expansion and pyroelectric effects [12,13]. Sub-microsecond responses have been demonstrated, enabling the scaling of such effects toward photonic integrated circuits with minimal coupling losses [14]. For instance, the *Q_m_* of commercial macroscopic PZT devices and MEMS-based piezoelectric structures differ significantly (≈85 [15] and ≈2857 [16], respectively). This difference arises from miniaturization effects, where reduced cavity dimensions enhance mechanical confinement and energy conversion efficiency. Moreover, MEMS devices benefit from integrated optical coupling and reduced damping losses, whereas macroscopic PZTs exhibit higher inertial mass and larger mechanical dissipation, leading to lower resonance sharpness and activation efficiency. These activation mechanisms are finding applications in fields such as medicine, adaptive optics, health monitoring, and intelligent remote sensing systems [17]. However, comprehensive experimental analyses that simultaneously compare electrical, mechanical, and optical responses under frequency-mode excitation remain scarce.

Addressing this gap, this work introduces an experimental approach that combines impedance spectroscopy, optical interferometry, and pulsed-laser activation to investigate the dynamic behavior of commercial PZT devices. The study aims to correlate resonance–antiresonance features with interferometric strain measurements, while also exploring laser-induced activation mechanisms. By examining both piezoelectric and pyroelectric contributions, this work provides new insights into non-invasive, frequency-mode optical excitation of piezoelectric devices and their potential for sensing and photonic integration.

## 2. Materials and Methods

### 2.1. Commercial Sensors

**Figure 1:**
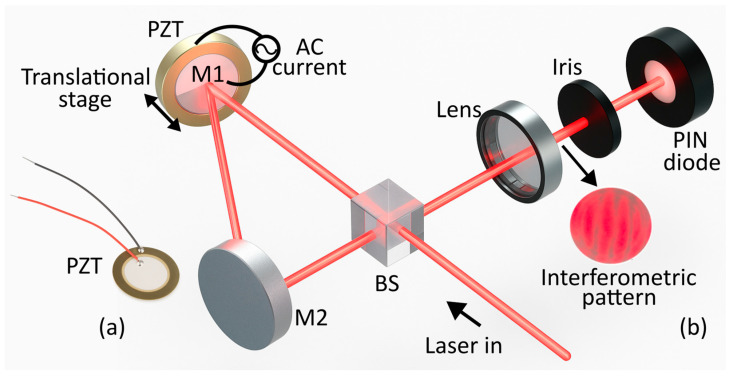
(**a**) Commercial actuators. (**b**) Sagnac interferometer setup.

We used a set of commercial piezoelectric actuators, Shenzhen Niannianxin Electronics Co., Shenzhen, China, with four diameters: 10 mm (S1), 12 mm (S2), 18 mm (S3) and 25 mm (S4). Each device is provided with two electrodes with specified electrical polarization (positive and negative) to enable induction and measurement of the piezoelectric effect. By design, the polarization direction is along the thickness axis. The sensors are shown in Figure 1a. The thickness of the samples was measured using a micrometer with a resolution of 5 µm. For these measurements, ten specimens per device diameter were considered, subtracting the brass layer to obtain the net PZT thickness.

### 2.2. Morphological and Elemental Characterization

The samples were studied by scanning electron microscopy (SEM) and energy-dispersive X-ray spectroscopy (EDS), using a ZEISS GeminiSEM 360 equipment, Carl Zeiss, Oberkochen, Germany. SEM analysis was used to determine the morphology of the piezoelectric devices and EDS analysis to define the materials involved in the samples.

### 2.3. Electrical Impedance Spectroscopy

The electrical impedance was measured using a 1010e Potentiostat system, Gamry Instruments, Warminster, PA, USA. The frequency of the input signal was swept from 10^2^ to 10^6^ Hz, with 1 V_p_ of amplitude. The data are presented in Bode-plot format to identify the resonance and antiresonance frequencies and to calculate the electromechanical coupling factors.

### 2.4. Stress–Strain Test

The actuators were mechanically characterized by using a standard stress–strain test using a universal testing machine model 3369, Instron Corporation, Norwood, MA, USA, with a load cell of 50 kN. The sensors were tested under compression in the elastic region to avoid damage with a displacement step of 1.6 µm. The Young’s modulus was obtained by linearly fitting the unitary displacement in the sample vs. the stress. The Young’s Modulus for the PZT devices was computed on 100 ± 5 MPa.

### 2.5. Interferometric Setup

The displacement caused by the piezoelectric effect under AC excitation was measured using a modified Sagnac interferometer (Figure 1b). A 638 nm, 4.8 mW continuous-wave laser, Thorlabs Inc., Newton, NJ, USA, was divided by a beam splitter, and both beams were reflected by mirrors (M1 and M2) along equal path lengths to create an interference pattern. The pattern was then filtered with an iris to reduce noise and detected by a FDS100 photodiode, Thorlabs Inc., Newton, NJ, USA. The electrical signal was recorded using an oscilloscope model 2190E, B&K Precision Co., Yorba Linda, CA, USA, in AC configuration to measure changes in the interferometric fringe displacement and remove DC components. The piezoelectric sensor was mounted behind M1, and a 5 Vp variable-frequency AC source was used for excitation. Mirror displacement—calibrated via a micrometric stage—was detected as fringe variations, correlating optical signals with mechanical deformation. Each measurement was repeated ten times per sample.

### 2.6. Pyroelectric Effect Measurement

The pyroelectric effect was measured by simultaneously recording the induced piezoelectric voltage and the temperature change under laser irradiation. A quasi-continuous 1064 nm laser, Laserglow Technologies, Toronto, ON, Canada, with nanosecond pulse duration was used. The laser emitted a pulse train within the 2.2–19 kHz range, with power modulated between 15 and 93 mW. The pulse width was 27 ns, the spot diameter 1.2 mm, and the mean energy per pulse 6.3 ± 0.4 µJ, slightly decreasing as the repetition rate increases. The pulse energy was approximately 6.7 µJ at 2.2–10 kHz and gradually decreased to 5.4 µJ at 19 kHz. The piezoelectric voltage was acquired in parallel with the electrodes using an oscilloscope, while the photothermal response was measured with an infrared sensor (0.2 °C resolution and 0.1 s sampling time). Both measurements were time-synchronized.

## 3. Theory

### 3.1. Equivalent Electromechanical Circuit

**Figure 2:**
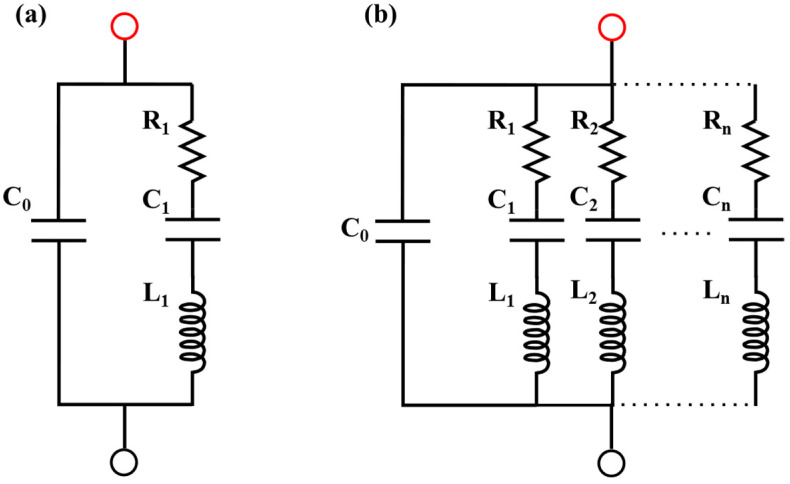
Equivalent piezoelectric circuit: (**a**) Fundamental frequency, (**b**) harmonic frequencies. Negative and positive electrical contacts are indicated by black and red points, respectively. The dotted line represents the n-circuits that may exist.

The electrical impedance of the sample was modeled using the Van Dyke equivalent circuit [15], which consists of a parallel configuration of a capacitor and a series RLC (resistor-inductor-capacitor) branch (see Figure 2a). The transfer function of the equivalent circuit is derived by considering the individual element impedances $Z_{C}={\left(Cs\right)}^{-1}$ for the capacitors, and $Z_{L}=Ls$ for the inductors, and the equation is expressed as:

(1) $$Z\left(s\right)=\frac{1}{C_{0}s}||\left(L_{1}s+\frac{1}{C_{1}s}+R_{1}\right)=\frac{1}{C_{0}s}||\frac{{C_{1}L}_{1}s^{2}+R_{1}C_{1}s+1}{C_{1}s}$$

where *Z*(*s*) is the equivalent impedance of the piezoelectric device, computed as the parallel of *C*_0_ with the series of *L*_1_, *C*_1_, and *R*_1_. Since the sample behaves as a resonant medium that supports harmonic frequencies determined by its geometry, several resonances are present in the electrical response. To model the normal modes of vibration can be considered an additional parallel RLC circuit can be included for each harmonic mode. A more complete equivalent circuit is shown in Figure 2b.

The subscript of each element denotes the number of harmonic resonances considered in the study. By adding *n*-elements RLC circuits in parallel, the impedance transfer function can be expressed as:

(2) $$Z\left(s\right)=\frac{1}{C_{0}s}||\frac{{C_{1}L}_{1}s^{2}+R_{1}C_{1}s+1}{C_{1}s}||\frac{{C_{2}L}_{2}s^{2}+R_{2}C_{2}s+1}{C_{2}s}||\ldots ||\frac{{C_{n}L}_{n}s^{2}+R_{n}C_{n}s+1}{C_{n}s}$$

Considering the *i*-th RLC circuit defined by the elements *L_n_*, *C_n_,* and *R_n_*, a general form of the transfer function can be written as:

(3) $$Z_{i}=\frac{{C_{i}L}_{i}s^{2}+R_{i}C_{i}s+1}{C_{i}s}=\frac{a_{i}}{b_{i}}$$

Therefore, the parallel result of *n*-th RLC circuits can be expressed as:

(4) $$Z_{1}||\ldots ||Z_{n}={\left[\sum_{i=1}^{n}\frac{1}{Z_{i}}\right]}^{-1}={\left[\sum_{i=1}^{n}\frac{b_{i}}{a_{i}}\right]}^{-1}=\frac{a_{1}a_{2}\ldots a_{n}}{b_{1}a_{2}a_{3}\ldots a_{n}+b_{2}a_{1}a_{3}\ldots a_{n}+\cdots +b_{n}a_{1}a_{2}\ldots a_{n-1}}$$

Writing in a general form:

(5) $$Z_{1}||\ldots ||Z_{n}=\frac{\prod_{i=1}^{n}a_{i}}{\sum_{i=1}^{n}\left[b_{i}\prod_{\begin{array}{c} j=1\\ j\neq i \end{array}}^{n}a_{j}\right]}=\frac{\prod_{i=1}^{n}\left({C_{i}L}_{i}s^{2}+R_{i}C_{i}s+1\right)}{\sum_{i=1}^{n}\left[C_{i}s\prod_{\begin{array}{c} j=1\\ j\neq i \end{array}}^{n}\left({C_{j}L}_{j}s^{2}+R_{j}C_{j}s+1\right)\right]}$$

Finally, the equivalent impedance of *n*-th RLC elements and the global capacitance *C*_0_ is given by:

(6) $$Z\left(s\right)=\frac{1}{C_{0}s}||\frac{\prod_{i=1}^{n}\left({C_{i}L}_{i}s^{2}+R_{i}C_{i}s+1\right)}{\sum_{i=1}^{n}\left[C_{i}s\prod_{\begin{array}{c} j=1\\ j\neq i \end{array}}^{n}\left({C_{j}L}_{j}s^{2}+R_{j}C_{j}s+1\right)\right]}$$

### 3.2. Piezoelectric Description

The mechanical stress along the poling axis of a polarized piezoelectric transducer is given by [11]:

(7) $$\sigma =\frac{{(\varepsilon }_{r}-1)\varepsilon_{0}}{d_{33}\tau }V$$

where *V* is the applied voltage, τ is the sample thickness, $\varepsilon_{r}$ the relative permittivity, $\varepsilon_{0}$ the vacuum permittivity, *σ* is the stress, and *d*_33_ is the piezoelectric coefficient. Considering the Young Modulus relationship σ=Es, where s=∆t/t is the axial strain, the piezoelectric deformation (∆t) can be expressed as a function of the Young Modulus as:

(8) $$∆t=\frac{(\varepsilon_{r}-1)\varepsilon_{0}}{d_{33}E}V=\frac{\chi_{e}\varepsilon_{0}}{d_{33}E}V$$

Furthermore, the deformations of a piezoelectric device subjected to an AC electrical input exhibit time-dependent behavior, associated with the relative permittivity obtained for the sample impedance [11]:

(9) $$\varepsilon_{r}(\omega )=-\frac{1}{\varepsilon_{0}}\cdot \frac{Z\prime \prime }{{Z\prime }^{2}+{Z\prime \prime }^{2}}\cdot \frac{\tau }{\omega A}$$

where *Z*′ and *Z*″ are the real and imaginary parts of the impedance, *ω* is the angular frequency, and *A* is the electrode area of the sample. By combining Equations (8) and (9), ∆t can be expressed as an electrical frequency-dependent quantity.

Additionally, electromechanical parameters were computed to evaluate the performance of the piezoelectric devices. The mechanical quality factor ($Q_{mi}$) of the *i*-th LRC circuit was calculated from the equivalent electrical components of the impedance spectrum as [18]:

(10) $$Q_{mi}=\frac{1}{R_{i}}\sqrt{\frac{L_{i}}{C_{i}}}$$

Likewise, the electromechanical coupling factor $k_{effi}$ can be obtained from the resonance $\left(f_{r}\right)$ and antiresonance $\left(f_{a}\right)$ frequencies as:

(11) $$k_{effi}=\sqrt{\frac{{f_{ai}}^{2}-{f_{ri}}^{2}}{{f_{ai}}^{2}}}$$

where the resonance and antiresonance frequencies are determined by:

(12) $$f_{ai}=\frac{1}{2\pi }\sqrt{\frac{C_{0}+C_{i}}{C_{0}C_{i}L_{i}}}$$

(13) $$f_{ri}=\frac{1}{2\pi \sqrt{C_{i}L_{i}}}$$

These parameters provide direct insight into the energy conversion efficiency, dynamic response, and mechanical losses of the system. These factors are critical for the design and application of sensors, actuators, and piezo-optical systems operating in the frequency domain.

## 4. Results

**Figure 3:**
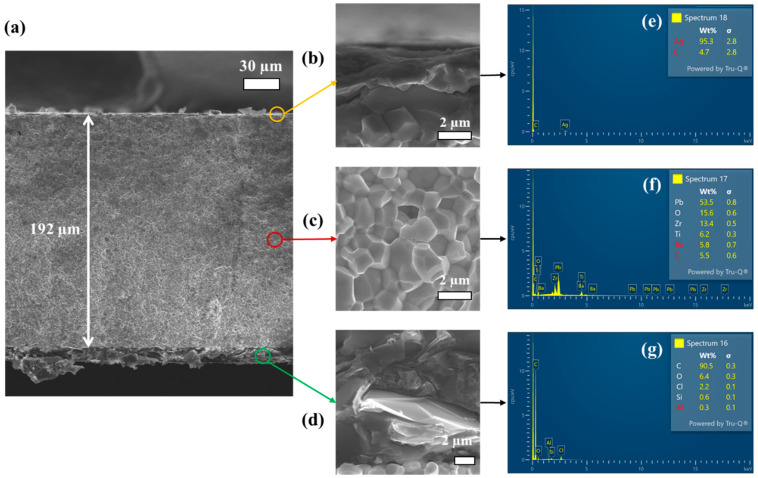
SEM analysis. (**a**) Far view of the PZT device. Zoom view of (**b**) Ag coating, (**c**) PZT grains, and (**d**) Carbon interface (scale bar 2 µm). EDS analysis (**e**) Ag coating, (**f**) PZT grains, and (**g**) Carbon interface.

To better understand the internal structure and material composition, one of the commercial PZT sensors was cross-sectioned and analyzed by SEM and EDS. This characterization aimed to identify structural components relevant to its electromechanical performance. As shown in Figure 3a, the cross-sectional SEM image revealed three distinct layers: a top silver (Ag) electrode (~3 µm thick), a central piezoelectric ceramic layer (~192 µm), and a bottom carbon-based layer (~13 µm) providing mechanical flexibility. Although SEM analysis was only performed in the S4, all samples are expected to share a similar composition, as they are mass-produced. The thickness of forty specimens (ten per diameter) was measured, yielding an average PTZ thickness of 193.8 ± 8 µm after subtracting the carbon and silver layers (~16 µm). This result indicates that, although the samples are not perfectly homogeneous, they all exhibit very similar thickness values. Higher-magnification images (Figure 3b–d) show that the piezoelectric layer displays a granular microstructure, likely resulting from powder metallurgy using PZT-based powders. Grain size analysis revealed values ranging from 1–4 µm, with a mean value of 1.9 ± 0.7 µm. EDS analysis confirmed the elemental composition of the layer (see Figure 3e–g). Figure 3e confirms the presence of Ag in the electrode, Figure 3f identifies Pb, Zr, Ti, and O as the main constituents of the PZT ceramic; and Figure 3g confirms carbon in the bottom layer. This carbon layer, in contact with the metallic substrate, is expected to enhance mechanical compliance and serve as an ohmic interface, thereby improving strain transfer and reducing electrical noise [19].

**Figure 4:**
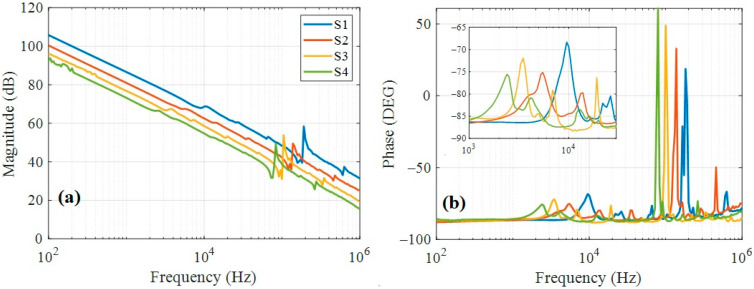
Bode plot of the four piezoelectric devices. (**a**) Magnitude and (**b**) phase. Inset is a zoom between 1 kHz and 30 kHz.

The electrical-impedance characterization of the four piezoelectric devices is shown in Figure 4 as a Bode diagram. The impedance magnitude, computed as $M_{dB}=20log(Z)$, exhibits the typical piezoelectric resonance and antiresonance features [20], as shown in Figure 4a. The magnitude curves have similar slopes but different phases, associated with the global capacitance of the devices, which scales proportionally with the electrode area. Additionally, strong resonances are observed near 10^5^ Hz, which increase in frequency as the device diameter decreases. Figure 4b shows the corresponding phase diagram, revealing that the resonance peaks shift to lower frequencies for larger devices. The main resonance peaks occur at 182 kHz, 138 kHz, 100 kHz, and 79.5 kHz for the S1, S2, S3, and S4 devices, respectively. Moreover, resonant peaks in the audible range also exhibit a monotonic frequency shift, appearing at 9.5 kHz, 5.5 kHz, 3.5 kHz, and 2.4 kHz for the same sequence of devices. These resonance behaviors were subsequently used to analyze electromechanical characteristics and to propose potential engineering applications.

**Figure 5:**
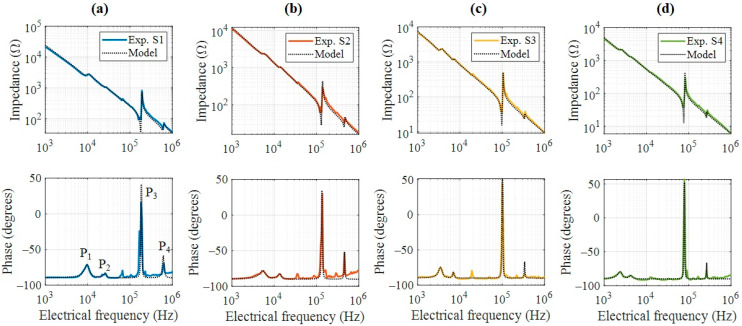
Bode plot of the piezoelectric devices and its numerical model. (**a**) S1, (**b**) S2, (**c**) S3, and (**d**) S4 samples.

To provide a deeper analysis of the impedance response, the experimental data were fitted using the electromechanical coupling model of the circuit shown in Figure 2b and described in Section 3.1 (Equation (6)). The curves were fitted using four peaks (P1, P2, P3, and P4), as shown in Figure 5, which are the more representative ones and are present in Figure 5. Figure 5a–d represent the samples S1, S2, S3 and S4, respectively. Since the sample thicknesses are similar, these peaks are attributed to radial vibration modes of the PZT disks, given that thickness modes typically appear at frequencies above 1 MHz for thin specimens [21]. The electrical characterization of the piezoelectric devices through impedance spectroscopy exhibited excellent agreement with the theoretical response expected for resonant systems. By fitting the impedance curves to the standard Van Dyke equivalent circuit model, it was possible to accurately extract the electrical and mechanical parameters of the samples, including the resonance and antiresonance frequencies, mechanical quality factor, and coupling factor. This modeling not only confirms the piezoelectric behavior in frequency mode but also provides a foundation for correlating it with interferometric displacement measurements and for estimating intrinsic material constants such as the piezoelectric coefficient. Table 1 summarizes the dimensions and global capacitance fitting results derived from the experimental data. The relative permittivity was calculated from $\varepsilon_{r}=C_{0}{\tau /\varepsilon }_{0}A$, yielding values between 1237 and 1278, in good agreement with PZT ceramics materials [22].

Based on the fitted electrical elements obtained from the experimental data, the mechanical quality factor, and the electromechanical coupling factor were calculated for the four representative resonance peaks. Equations (10)–(12) were applied, and the results are summarized in Table 2. The quality factor $Q_{m}$ increases significantly with resonance order in all samples, reaching values above 40 in higher modes, which indicates reduced mechanical losses at high frequencies. In the first resonance $Q_{m1}$ was approximately 2 and 4 in all samples—a relatively low value compared with that of dense or highly optimized PZT ceramics [23,24]. This reduced value is consistent with the granular microstructure observed in SEM analysis and suggests significant internal mechanical losses, as expected in low-density piezoelectric materials [25]. Furthermore, the effective coupling factor $k_{eff}$ exhibits peak values in modes 1 and 3, particularly in sample S1, which demonstrates the best overall electromechanical performance.

Subsequently, the piezo-optical effect and the deformation behavior were evaluated in the low-frequency (acoustic) range to access directly the dominant vibrational modes responsible for macroscopic strain, which are the most relevant for interferometric detection. Although higher-frequency modes (e.g., in the hundreds of kHz range) exhibit higher $Q_{m}$ values, they are often associated with localized or high-order vibrational modes that do not produce measurable displacements over the entire device surface [26,27]. In contrast, the acoustic modes in the kHz range generate global deformation patterns that are more effectively captured by the interferometric setup, allowing for precise optical quantification of strain and direct correlation with the electromechanical response.

**Figure 6:**
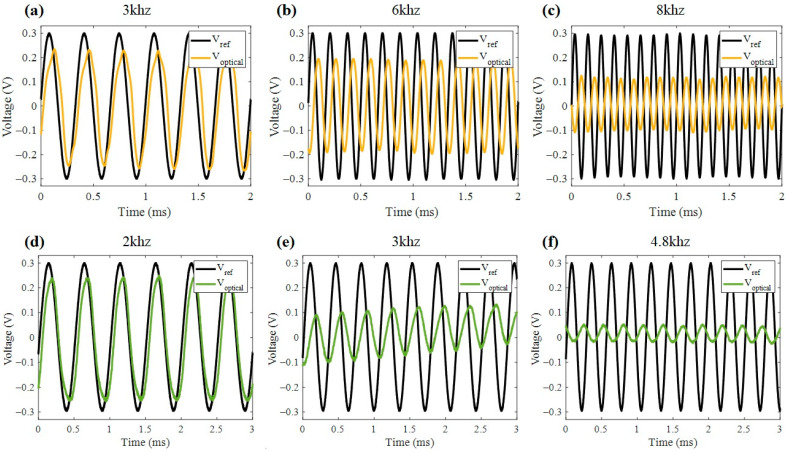
Optical signal reading by Sagnac interferometer at AC current input in samples: S3 (**a**) 3.0 kHz, (**b**) 6.0 kHz, and (**c**) 8.0 kHz; S4 (**d**) 2.0 kHz, (**e**) 3.0 kHz, and (**f**) 4.8 kHz.

Building on the previous findings, the strain response of the piezoelectric devices was measured using the modified Sagnac interferometer (Figure 1b), which was previously calibrated to establish a quantitative relationship between optical fringe displacement and mechanical deformation. The interferometric calibration (performed using a micrometrical translational stage in mirror M1) provided a sensitivity of 20 µm/V, corresponding to the displacement measured by the photodiode. Each volt at the detector output represents an equivalent mirror displacement of 20 µm, while the actual driving voltage applied to the PZT remained within the linear regime. Subsequently, measurements were performed on two commercial PZT devices excited with an AC voltage signal swept across the acoustic frequency range (1 kHz to 10 kHz). For each sample, the interferometric setup recorded the dynamic displacement induced by the piezoelectric activation, allowing the extraction of strain information from the modulated interference fringes. Figure 6 shows the dynamic results of some representative frequencies specific to device S3 (Figure 6a–c) and S4 (Figure 6d–f). As a reference, the AC input voltage signal (*V_ref_*) applied to the electrodes is presented alongside the corresponding optical signal (*V_optical_*) detected in AC mode, which reflects the real-time deformation of the devices. The optical response shows clear frequency dependence, with maximum deformation occurring near the first mechanical resonance, in agreement with the impedance spectroscopy results.

**Figure 7:**
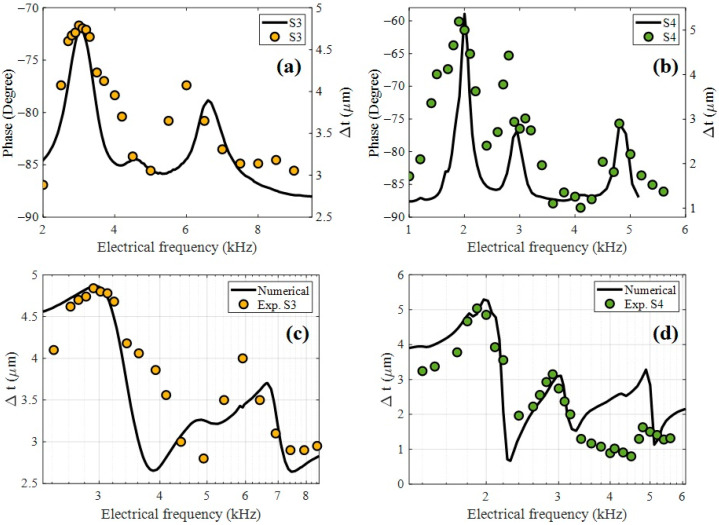
Impedance spectroscopy correlation of deformation and phase for samples (**a**) S3 and (**b**) S4. Comparison of numerical model of deformation with experimental data for samples (**c**) S3 and (**d**) S4.

Figure 7a and Figure 7b present a comparative analysis between the impedance phase response and the interferometric strain measurements in samples S3 and S4, respectively. These results reveal a strong correlation, particularly in the acoustic frequency range. The maximum deformation Δ*t* recorded by the interferometer (right axis) coincided with the frequency where the impedance phase exhibits a rapid transition, typically near the resonance peak. This behavior is consistent with the physical principle that, at resonance, the electromechanical coupling is maximized [28], leading to a peak in mechanical displacement and a characteristic phase shift in the electrical response. Furthermore, Figure 7c,d show the interferometric results compared with a numerical approximation based on a simplified electromechanical model of the device. The model shows good agreement with the experimental data, particularly around the dominant resonance frequency, validating both the optical calibration setup and the applicability of the equivalent circuit model for predicting dynamic strain behavior in frequency-mode operation. The piezoelectric coefficient *d*_33_ was estimated by correlating the measured strain amplitude with the model of Equations (8) and (9). The best fit to the data yielded a value of $d_{33}=40\;pC/N$, which is in the range of the typical reported values for granular PZT ceramics [29,30] and lower than those of dense bulk materials. This result confirms the sensitivity and reliability of the combined interferometric-electrical approach for quantitatively characterizing frequency mode-piezoelectricity.

**Figure 8:**
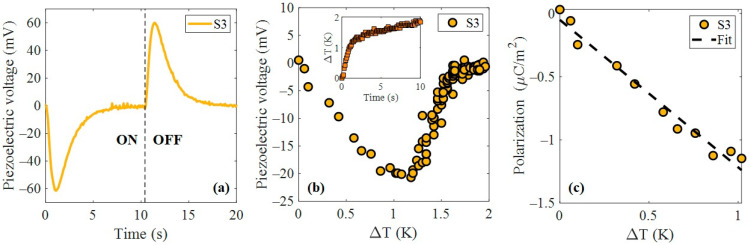
Piezo-optical response by kHz nanosecond laser source. (**a**) Piezoelectrical voltage induced by 15 kHz at 82 mW power in sample S3. (**b**) Pyroelectric effect in sample S3 by photothermal process. (**c**) Induced polarization by pyroelectric effect.

A piezo-optical activation experiment was conducted by irradiating the PZT devices with pulsed laser light to explore the generation of electrical signals via photo-induced mechanical stress. Sample S3 was tested using nanosecond laser pulses with a repetition rate in the kHz range. The optical pulse train coupled to the piezoelectric frequency modes of the sensor induced a mechanical deformation. The dynamic response at 15 kHz of frequency and 82 mW of power is present in Figure 8a (in which the laser was turned on for 10 s and off for another 10 s). A non-trivial behavior was observed in which the piezoelectric voltage became negative during laser irradiation. When the pump beam is turned off, the voltage exhibits a symmetric peak corresponding to the release of stored energy. The integral overtime was computed to be close to zero. This effect can be explained as a photothermal effect in which the sample expands due to its thermal expansion. The sample undergoes microscopic elongation opposite the poling axis, counteracted by the piezoelectric effect. The mechanical wave propagates along the surface, following both the wave equation and the heat-conduction equation [31].

To explore this nonlinearity, the pyroelectric effect was investigated as a contributing photothermal mechanism. Figure 8b shows the piezoelectric voltage as a function of device temperature, revealing a sudden change in output for a temperature variation of 1 K. The rapid temperature rise induces a polarization voltage, which gradually decays as the system approaches thermal equilibrium, even under continuous irradiation. The inset of Figure 8b shows the transient temperature change, which exhibits a steeper slope between 0 and 1.2 °C and decreases noticeably above 1.2 °C. This behavior confirms that the pyroelectric response depends on the rate of temperature change (dT/dt) rather than the absolute temperature, consistent with its transient polarization nature [32]. Accordingly, the induced polarization was estimated as $P=\left(\varepsilon_{r}-1\right)\varepsilon_{0}V/\tau$, yielding a pyroelectric coefficient of approximately −1.16 μC/m^2^K. This result, shown in Figure 8c, suggests that, in addition to direct piezoelectric activation, a thermally mediated polarization mechanism contributed to the optically induced electrical signal. In the model, the temperature dependence of εᵣ for PZT was neglected, as the operating range (T = 20 ± 2 °C) is insufficient to produce significant variations. While pyroelectric effects may be slightly affected, εᵣ(T) exhibits substantial changes only above approximately 200 °C, where it follows an exponential behavior [33,34].

After analyzing the piezoelectric, acoustic, and pyroelectric effects involved in the frequency-mode operation of the PZT-based system, we now propose the devices as potential instruments for engineering applications. Specifically, we explore its implementation as both a mass-sensing balance and an optical sensor, capable of modulating the phase and amplitude of an AC signal at the maximum impedance peak. By tracking the phase shift and magnitude variation at this resonant condition, the system enables the quantification of either mass loading or laser power. In the case of the mass balance, the resonance frequency shift correlates with added mass, while in the optical configuration, the incident pulsed laser induces piezo-optical interactions that alter the impedance profile.

**Figure 9:**
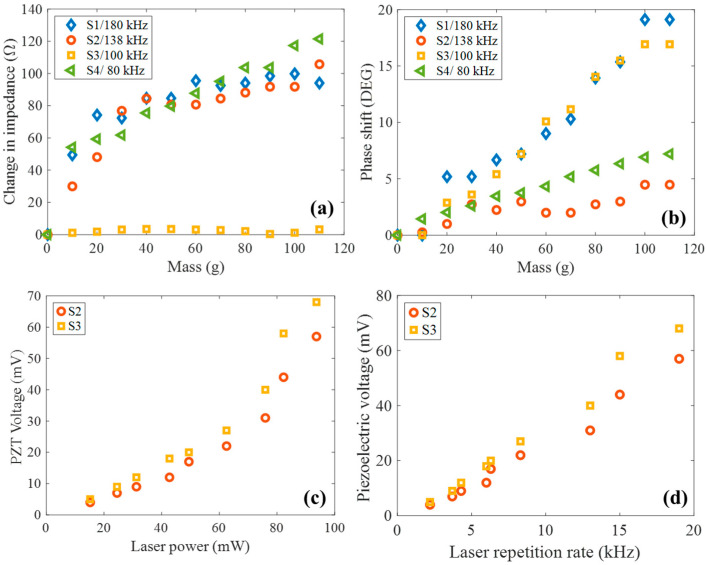
Devices implemented as frequency-mode mass balances. (**a**) Change in impedance and (**b**) phase shift. Piezo-optical voltage in samples S2 and S3 as a function of (**c**) laser power and (**d**) laser repetition rate.

Figure 9a and Figure 9b show the change in impedance and phase shift, respectively, of the devices by loading controllable masses. The samples were excited by AC current at the resonance frequency of peak P3 and subsequently measured their impedance and phase shift. The P3 resonance was selected because it showed the highest resonance magnitude and quality factor, indicating stronger electromechanical coupling and greater sensitivity to mass-induced perturbations. Samples S1, S2, and S4 exhibit similar changes in impedance trends, reaching approximately 80 Ω at 50 g. Additionally, Sample S4 exhibits a quasi-linear trend from 10 g to 110 g range. Sample S3 maintained an almost constant impedance across the entire mass range, indicating minimal variation in its electrical response under load. Furthermore, the phase shift in samples S2 and S4 does not change drastically throughout the entire range, indicating low sensitivity to mass-induced perturbations in phase. Samples S1 and S3 presented an increasing behavior in phase shift with mass, reaching approximately 18°. These observations highlight a varying degree of sensitivity and mass-loading response among the samples, with phase shift being a particularly distinctive parameter. Notably, the differences in phase and magnitude responses do not scale proportionally with diameter, suggesting that geometrical factors and mechanical wave propagation contribute in a non-trivial way to the electromechanical behavior of each device. The resonant modes of the piezoelectric disks are governed by boundary conditions, which are changed by the addition of mass at the center. This modification directly influences the frequency modes, which can become either constructive or destructive depending on how the added mass perturbs the resonant cavity dynamics.

Additionally, the sensors used as optical detectors proved to be effective for measuring both the power and repetition rate of a laser. Due to their pyroelectric properties, they could determine the laser power as its repetition rate increased within the acoustic range (2–20 kHz). Figure 9c shows the output voltage, which exhibited nonlinear dependence as a function of optical power, suggesting possible contributions from thermomechanical or multiphoton effects at higher intensities. The results of Figure 9d demonstrated that the maximum voltage generated by the device increased linearly with the laser repetition rate. This behavior consists of a repetition rate-dependent accumulation of charge due to the dynamic pyroelectric effect, where periodic temperature changes induced by laser pulses lead to voltage generation across the material. Only the results for samples S2 and S3 are shown, as the other devices produced similar values that obscured comparative visualization. This dual-functionality opens the path for compact, low-cost, frequency-selective sensors with potential in real-time diagnostics and integrated optomechanical instrumentation.

## 5. Discussion

The present study provides an integrated understanding of how piezoelectric and optically induced behaviors can be analyzed through frequency-domain measurements of commercial PZT devices. Through a combination of electrical impedance measurements, optical interferometry, and piezo-optical activation, a clear relationship was established between mechanical deformation and both the electrical and optical responses of the samples. The resonant and antiresonant behavior agreed well with the predictions of classical piezoelectric vibration theory. It was established that all observed vibration modes (>1 MHz) correspond to radial modes. Since the thickness is constant and no resonance appears at the same frequency, thickness vibration modes were not detected [28]. The behavior of vibration depends mainly on the disk diameter, as the radius is much larger than the thickness, and the motion is governed by boundary conditions through Poisson’s ratio [35]. Although slight variations in electrode thickness and mass loading could influence the resonance frequencies, their effect is negligible. The silver electrode mass (1–7 × 10^−4^ kg) exerts an almost constant pressure of about 1.54 Pa in the devices, far below the kilopascal range known to cause measurable frequency shifts [15]. As all samples share identical clamping and fabrication conditions, the resonance frequency primarily varies with the disk diameter, which defines a cavity with a variable wavenumber ($k_{T}$) following: $f=k_{T}C_{T}/2\pi$, where $C_{T}$ is the wave speed.

Furthermore, the optically induced voltage response, particularly under modulated laser excitation, suggests the presence of dynamic pyroelectric effects that became significant at higher frequencies and power levels. These observations suggest that, beyond traditional electrical excitation, optical signals can serve as reliable tools to probe and even activate piezoelectric materials under controlled conditions. This approach lies in the synergistic integration of impedance spectroscopy and interferometric analysis under frequency-mode excitation, enabling the simultaneous and correlated evaluation of electrical and mechanical responses in a single piezoelectric element. Unlike conventional characterization methods that typically probe each domain separately, this hybrid methodology establishes a direct correlation between impedance phase and interferometric deformation, providing a self-referenced and frequency-resolved signature of the device’s electromechanical dynamics. This integration allows non-contact, frequency-selective activation and quantitative assessment of piezoelectric and pyroelectric contributions, thereby defining a unified, non-invasive, and scalable platform for future sensing and optoelectromechanical applications.

The macroscopic evaluation of commercial PZT discs offers relevant insight into photo-piezoelectric interactions that are fundamentally governed by optical and electromechanical coupling. Because these effects originate from intrinsic material responses, such as photoinduced polarization, pyroelectricity, and elastic-wave propagation, their principles can be considered applicable across different dimensional scales. Previous studies have demonstrated that PZT thin films and nanostructured ferroelectrics preserve consistent polarization and piezoelectric behavior under optical and electrical excitation [36,37,38]. Moreover, MEMS and NEMS platforms based on PZT and related materials have exploited similar mechanisms for energy harvesting, sensing, and photonic modulation [39]. Therefore, while this work focuses on macroscopic devices, its optical activation framework provides a conceptual link to smaller-scale implementations where analogous effects can be further explored.

From a broader perspective, operating in the frequency domain opens new possibilities for considering how piezoelectric materials might be integrated into biomedical platforms [40,41]. The close match between impedance phase, deformation, and optically generated voltages points to the feasibility of developing piezoelectric systems that respond to external light rather than mechanical loads, which can be used for applications like tissue regeneration or neurostimulation [42,43]. While this work was carried out using lead-based ceramics, the same approach could be extended to biocompatible materials such as barium titanate or hydroxyapatite, which combine functional piezoelectricity with bioactivity [41,44]. One particularly promising path is to explore nonlinear optical mechanisms, like two-wave mixing, in these materials to induce localized electric gradients that could influence cellular behavior without mechanical contact [45]. Overall, the insights from this work help define a path toward designing responsive piezoelectric microstructures in which optical signals serve as both stimuli and control parameters for microscale non-invasive therapeutic devices [46].

## 6. Conclusions

This work presented a comprehensive experimental framework for characterizing the frequency-dependent electromechanical behavior of commercial piezoelectric (PZT) devices, combining electrical impedance spectroscopy, interferometric displacement measurements, and pulsed-laser activation. Morphological and elemental analyses confirmed the granular nature of the PZT samples, which exhibited low mechanical quality factors and stiffness, characteristics typical of ceramic-based composites. Key parameters such as resonance and antiresonance frequencies, effective stiffness, and piezoelectric coefficients were extracted and validated through agreement between impedance phase shifts and dynamic deformation captured via optical interferometry. The response to pulsed-laser excitation revealed an inverse voltage output, indicating contributions from both piezoelectric and pyroelectric mechanisms, with a pyroelectric coefficient of −1.16 μC/m^2^·K estimated from temperature-dependent measurements.

While the study focused on macroscale commercial devices, the methodologies developed—non-contact optical readout, frequency-mode resonance analysis, and optically triggered activation— are inherently scalable. These tools could be adapted for thin-film and nanowire-based systems, particularly where conventional electrical contacts can modify the measured properties. Moreover, the observed laser-power and frequency-dependent responses highlight potential applications in micro- and nanoscale sensing platforms, such as mass-balance resonators or optical power meters. As such, this work provides foundational techniques that can inform the design, testing, and optimization of miniaturized piezoelectric devices. Future research should focus on integrating these methods with nanoscale-specific modeling and fabrication strategies to enable precise control and characterization at reduced dimensions.

## Figures and Tables

**Table 1:** Morphological and dielectric characteristics of samples.

Sample	Ø (mm)	A (mm^2^)	C_0_ (nF)	*ε_r_*
S1	10	78.5	4.5	1243
S2	15	176.7	9.5	1277
S3	18	254.4	16	1237
S4	25	490.8	24	1278

**Table 2:** Piezoelectric coupling parameters. *f_ri_*, *f_ai_*, *Q_mi_*, and *k_effi_* refer to the computed parameter of the *i*-th peak.

Sample	P1				P2				P3				P4			
	*f_r_*_1_	*f_a_*_1_	*Q_m_*_1_	*k_eff_*_1_	*f_r_*_2_	*f_a_*_2_	*Q_m_*_2_	*k_eff_*_2_	*f_r_*_3_	*f_a_*_3_	*Q_m_*_3_	*k_eff_*_3_	*f_r_*_4_	*f_a_*_4_	*Q_m_*_4_	*k*_eff4_
	(kHz)				(kHz)				(kHz)				(kHz)			
S1	9.5	10.1	2.8	0.35	25.4	25.8	4.8	0.16	178.8	191.7	32.5	0.36	601.5	618.8	9.44	0.23
S2	5.3	5.6	2.2	0.31	13.4	13.7	4.2	0.18	130.6	139.1	30.1	0.34	459.4	466.3	23.1	0.17
S3	3.4	3.5	3.9	0.27	6.9	7.0	10.9	0.12	97.9	104.6	42.3	0.35	336.3	339.0	24.1	0.12
S4	2.3	2.4	3.1	0.25	4.2	4.3	4.6	0.13	76.8	81.5	48.3	0.33	265.2	267.1	30.3	0.11

## Data Availability

Data available on request from the authors.
